# Evidence for cervical cancer mortality with screening program in Taiwan, 1981–2010: age-period-cohort model

**DOI:** 10.1186/1471-2458-13-13

**Published:** 2013-01-08

**Authors:** Shih-Yung Su, Jing-Yang Huang, Chien-Chang Ho, Yung-Po Liaw

**Affiliations:** 1 Department of Public Health and Institute of Public Health, Chung Shan Medical University, No. 110 Sec 1 Chien-Kuo N. Road, Taichung City 40201, Taiwan; 2 Institute of Epidemiology and Preventive Medicine, College of Public Health, National Taiwan University, Rm. 536, No. 17, Xuzhou Rd, Taipei 100, Taiwan; 3 Department of Health and Leisure Management, Yuanpei University, Hsinchu City, 30015, Taiwan

**Keywords:** Cervical cancer, Age-period-cohort model, Mortality, Gynecologic oncology, Screening program

## Abstract

**Background:**

Cervical cancer is the most common cancer experienced by women worldwide; however, screening techniques are very effective for reducing the risk of death. The national cervical cancer screening program was implemented in Taiwan in 1995. The objective of this study was to examine and provide evidence of the cervical cancer mortality trends for the periods before and after the screening program was implemented.

**Methods:**

Data from 1981 to 2010 of the causes of death registered were obtained from the Department of Health, Taiwan. Age-standardized mortality rates, age-specific rates, and age-period-cohort models that employed the sequential method were used to assess temporal changes that occurred between 1981 and 2010, with 1995 used as the separating year.

**Results:**

The results showed that for both time periods of 1981 to 1995 and 1996 to 2010, age and period had significant effects, whereas the birth cohort effects were insignificant. For patients between 80 and 84 years of age, the mortality rate for 1981 to 1995 and 1996 to 2010 was 48.34 and 68.08. The cervical cancer mortality rate for 1996 to 2010 was 1.0 for patients between 75 and 79 years of age and 1.4 for patients between 80 and 84 years of age compared to that for 1981 to 1995. Regarding the period effect, the mortality trend decreased 2-fold from 1996 to 2010.

**Conclusions:**

The results of this study indicate a decline in cervical cancer mortality trends after the screening program involving Papanicolaou tests was implemented in 1995. However, the positive effects of the screening program were not observed in elderly women because of treatment delays during the initial implementation of the screening program.

## Background

Cervical cancer is the most common and serious gynecologic malignancy worldwide. This finding highlights the urgent need to significantly increase cancer survival rates and reduce mortality through screening programs. Cervical screening methods that involve cytology tests, the Papanicolaou technique (PT), human papillomavirus (HPV) tests, and visual inspections of the cervix with 5% acetic acid (VIA) have been established to reduce the cervical cancer mortality rate by almost 60% in numerous countries [1,2]. National cervical cancer screening programs have been implemented in many countries throughout the world, including Australia [3], Nordic countries (that is, Iceland [4], Scandinavia [5], Finland [6], and Norway [7]), England [8], France [9], Germany [10], the U.S. [11], and Canada [12]. Additionally, many recent studies have shown that the efficacy of screening programs have increased the survival rate and reduced the mortality rate of cervical cancer.

According to the World Health Organization (WHO)’s cervical cancer screening guidelines, screening is defined as testing all women at risk of cervical cancer, most of whom will not exhibit symptoms. The objective of screening is to detect precancerous changes that, if left untreated, can lead to cancer. However, screening is only effective if a well-organized follow-up and treatment system is also provided. Women who are found to have abnormalities during screening require follow-up consultations, diagnosis, and possibly treatment to prevent the development of cancer or to treat cancer in the initial stage. Several tests can be used to screen for cervical cancer. However, the Papanicolaou smear (Pap cytology) is the only test that has been applied to large populations and been shown to reduce the incidence and mortality rate of cervical cancer. Although VIA, and HPV test have shown potential, to date, no comparable evidence of their effectiveness exists. Large-scale studies are still being conducted. Regardless of the test used, the key to an effective program is to target the largest proportion of women at risk with quality screening and treatment. Organized screening programs that are designed to reach most women at risk and managed at a central level are preferable to opportunistic screening.

The Bureau of Health Promotion of the Department of Health implemented the screening program in Taiwan in 1995. Under the screening policy, free annual medical examinations are provided to all Taiwanese women aged 30 years or older. Women who are sexually active are strongly advised to undergo a smear test at least once every 3 years. Women who have been diagnosed with a sexually transmitted disease, the human immunodeficiency virus, the human papillomavirus, or cervical dysplasia, and who have numerous sexual partners, should undergo testing once every year. The results of a smear test provide a reference for whether further examinations are necessary. If the test results are unclear regarding pathological changes, women are recommended to undergo a biopsy. If the biopsy indicates carcinoma in situ, women will only require a simple operation to fully cure the disease.

Through a time trend analysis of mortality rates, we can evidence the efficacy of cervical screening programs. The age-period-cohort (APC) model is one of the most common statistical methods. In a previous study [13], a number of researchers presented the cervical cancer mortality trends in Taiwan; however, that study analyzed mortality trends between 1974 and 1992. Therefore, the aim of this study was to examine the cervical cancer mortality rates following the implementation of the screening program, and identify differences that occurred around the time the screening program was implemented.

## Methods

Data from 1981 to 2010 of the causes of death registered were obtained from the Department of Health, Taiwan. We selected three variables for sequentially analysis, that is, age of death, period of death, and the coding of International Classification of Diseases (ICD). To determine the three factors (age, period, and cohort) for time trend analysis, we first excluded patients who were aged 19 and under and patients who were aged 85 and over before separating the participants into 13 groups of 5 age bands each. Second, we separated 6 aggregations by 5 years of death for each period group. Finally, cohort groups were calculated according to age groups and period groups. Cervical cancer was defined as malignancies of the endocervix (ICD-9^th^ for 180.0, and ICD-10^th^ for C53.0), exocervix (ICD-9^th^ for 180.1, and ICD-10^th^ for C53.1), other specified sites of the cervix (ICD-9^th^ for 180.8, and ICD-10^th^ for C53.8), and unspecified sites of the cervix uteri (ICD-9^th^ for 180.9, and ICD-10^th^ for C53.9).

The APC model that employs the sequential method [14] is a serial process that comprises two mathematical steps. First, we modeled the age effect and the period effect. Then the results are combined for the offset function to calculate the cohort effect in the second step. Finally, we also considered the model fit using the spline smoother function of the sequential method. We choose the natural spline function for modeling, and used the Akaike information criterion (AIC) to determine the appropriate number of knots. All age, period, and cohort effects were separated into two parts, those for 1981 to 1995 and those for 1996 to 2010. Additionally, we calculated the period ratios (1996 to 2010/1981 to 1995) according to age groups. We also determined the age-standardized mortality rate (ASMR) using the 2000 World Standard Population Report produced by the WHO as the reference population combined with the age specific rate for 30 years. All the statistical technics were conducted using R software.

## Results

**Figure 1:**
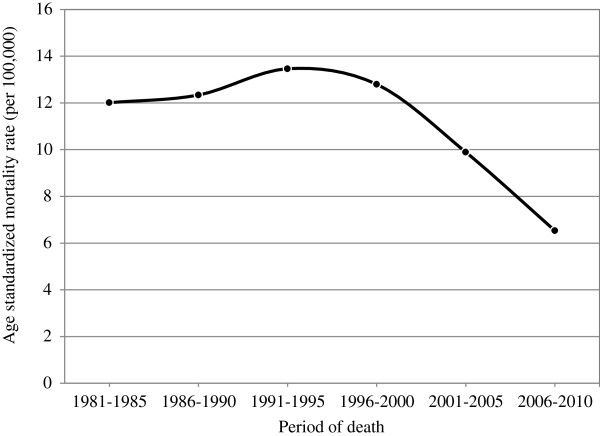
Age standardized mortality rates of cervical cancer in 1981–2010.

The ASMRs for cervical cancer from 1981 to 2010 are shown in Figure 1; the results show a watershed and indicate that the peak of the mortality trend reached 13.5 (per 100,000) in 1991 to 1995. However, the ASMRs declined by approximately 50% over the 30 years.

**Figure 2:**
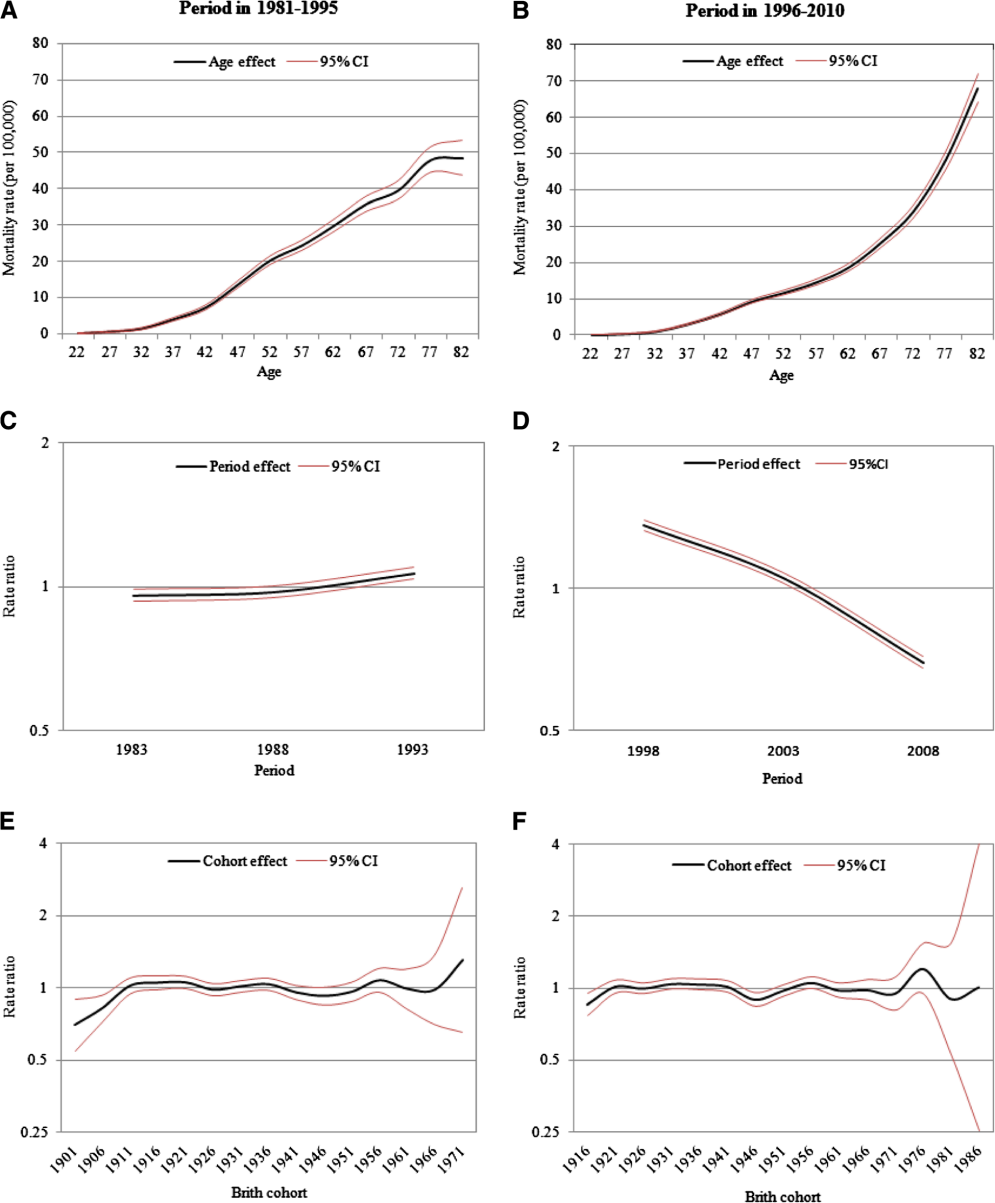
Age(A, B), period(C, D), and cohort(E, F) effects of cervical cancer between 1981–1995 and 1996–2010.

Figure 2 shows the age (A, B), period (C, D), and cohort (E, F) effects of cervical cancer for the two periods of 1981 to 1995 and 1996 to 2010. The results show that age had a significantly positive effect in each period, and the mortality rates for the oldest age group were 50 (per 100,000) and 68.1 (per 100,000) for 1981 to 1995 and 1996 to 2010. The period effects exhibited a contrary trend; that is, the mortality rate ratios gradually increased after 1981 before declining 2-fold from 1996 to 2010. No cohort effects were observed for both periods.

**Figure 3:**
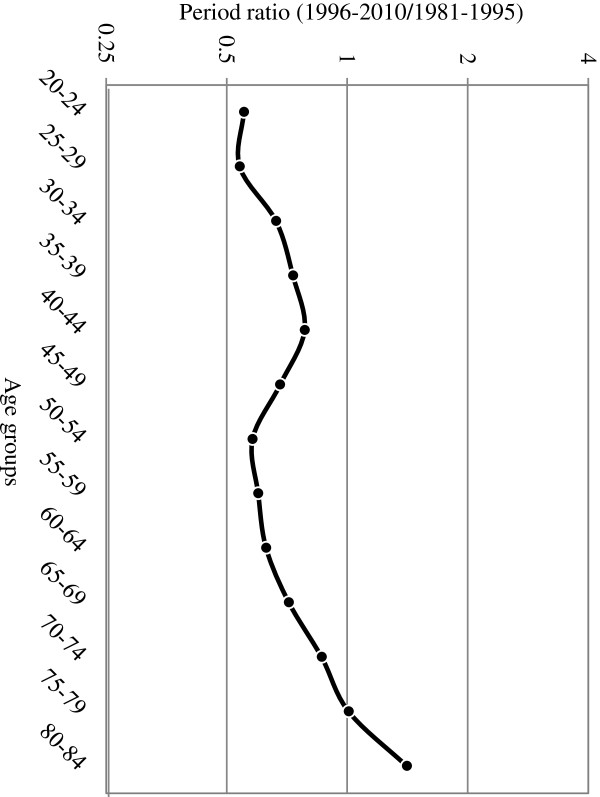
The differential period ratios of cervical cancer form age effects of age-period-cohort model.

The period ratio based on the age effect of the APC model is shown in Figure 3. The results show that the trend of increasing mortality rates in 1996 to 2010 compared to those for 1981 to 1995 was gradually augmented by age. Generally, the influence of the age effects on mortality rates after 1995 was lower than that before 1995 year. However, an equal level was exhibited by patients between 75 and 79 years of age, and patients between 80 and 84 years of age exhibited a ratio of 1.4-fold.

**Figure 4:**
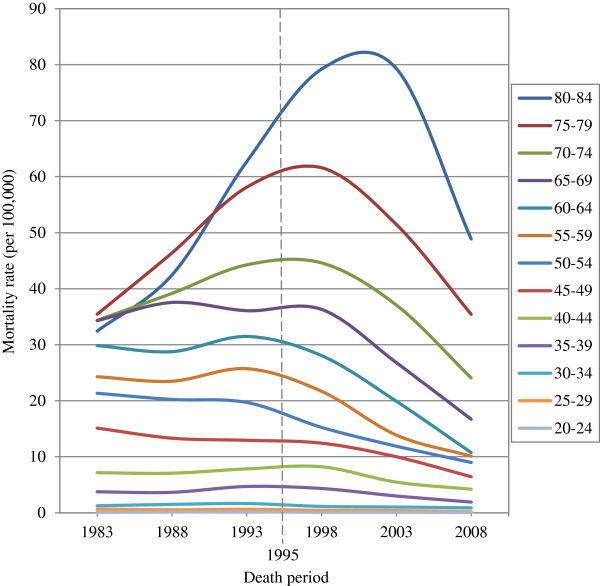
The age specific mortality rates of cervical cancer by each age group during 30 years.

The age specific mortality rates for cervical cancer according to each age group over 30 years are shown in Figure 4; the central dashed line denotes the year 1995. Each trend of mortality rates was similar to the ASMR. The results show that the peak of each mortality rate occurred around the dashed line before subsequently declining. However, the results indicated a lack of time for older patients between 75 and 79 years of age and 80 and 84 years of age. The age specific mortality rate of patients between 75 and 79 years of age and patients between 80 and 84 years of age decreased after 1998 until 2003.

## Discussion

We conducted separate APC model calculations for the periods of 1981 to 1995 and 1996 to 2010 to verify the hypothesis that the cervical screening program influenced the mortality trends after 1995. The results not only supported the positive effects of the screening program, but also showed unexpectedly patterns. First, the ASMR results and the period effects of the APC model both indicated that cervical cancer mortality trends were reduced after the screening program involving Papanicolaou test was implemented in 1995. Additionally, many previous studies have reported on the efficacy of the screening program in Taiwan [15-18]. Second, the effects of the screening program were not observed among elderly women.

The differential period ratios for cervical cancer show a decline in mortality rates for people between 30 and 79 years of age after the implementation of the screening program. By contrast, the risk of mortality caused by the protectiveness of the screening program was 1.4-fold higher than that during the periods before the screening program was implemented among patients between 80 and 84 years of age. These contradictory patterns may indicate a problem. Additionally, the screening rates among elderly women in Taiwan may be lower, preventing early diagnosis and treatment, thereby increasing their mortality rate and reducing their survival rate. However, the answer to this issue was found in the statistical data obtained from the Bureau of Health Promotion of the Department of Health. The data indicated that the screening rates were 55% to 65% for women aged between 30 and 39, 50% to 60% for women aged between 40 and 49, 45% to 55% for women aged between 50 and 59, 40% to 50% for women aged between 60 and 69, and 25% to 32% for women aged 70 or more. Thus, the screening rates were lower among elderly women.

The cervical cancer mortality rate for older age groups (70+) exhibited an increasing trend before 1995, but not among younger women. i.e. the mortality risks for women aged 70 years or more increased annually before 1995; whereas no significant change in mortality trends was observed among women under 70. Present studies cannot provide a suitable explanation for this finding. The reason for these results may be a particular risk factor directly or indirectly related to age that remains unknown. However, we still propose some possible reasons causing this phenomenon for subsequent study validation. Regarding the phenomenon of multiple births among women of the post-war generation, a number of previous studies have suggested that pregnancy at an excessively early age and excessive pregnancies may be risk factors for cervical cancer [19-22]. Taiwan experienced a post-World War II baby boom. Beginning in 1947, the birth rate increased from 38.31 to 49.97 by 1951. Consequently, we can infer that women who were 70 to 84 years of age between 1980 and 1995 were of a reproductive or childbearing age (i.e., 20 to 35 years of age) from approximately 1930 to 1960. This includes all women at childbearing age during the post-war baby boom.

However, the results of numerous previous studies also support the limited effectiveness of cervical cancer screening among elderly women [23-27]; one study even contended that screening elderly women was inefficient and should be terminated for women aged 65 or older who have a history of regular negative smears [27]. Considering this argument, we contend that if screening is genuinely ineffective among elderly women, then the mortality rate should remain unaffected. However, this is completely unreasonable in Taiwan. The pattern of age-specific mortality rates for each age group shown in Figure 4 exhibited a declining trend among elderly women. The results also indicated that the effectiveness of the screening program may not immediately affect women between 80 and 84 years of age in Taiwan because of treatment delays during the initial screening program implementation stage. Previous studies related to the effectiveness of screening have considered the problem of a lead time bias. However, the idea that reducing incidence was the main screening effect that could successfully reduce mortality rates was based on the notion that no deaths would be caused by a disease if the disease did not occur. However, the reduced mortality rate caused by screening is not entirely because of a lower or reduced incidence rate. Especially when cancer prognosis results are good, implementing screening may increase the incidence rate, but it can reduce mortality rates through early treatment. For example, in Taiwan, a few years after the screening program was implemented in 1995, the incidence of cervical cancer increased significantly; however, the corresponding mortality rate declined following the implementation of the screening program. Thus, the reduced mortality rate for cervical cancer cannot be mainly attributed to a lower incidence; instead, it was also affected by whether the Papanicolaou test provided good prognosis results, thereby increasing the patient survival rate.

In 2006, Lloroa [28] contended that the low cervical cancer mortality rates were not only the result of the Papanicolaou test or other screening techniques and treatment reforms, but could also be attributed to misclassifications on death certificates. The study showed that numerous uterine cancer cases were classified as “site unspecified.” According to Cuzick and Beral [29,30], uterine cancers can be classified into three types, that is, cervical cancer, corpus uteri cancer, and cancer of the uterus at an unspecified site (UNOS). However, Levi [31] believed that decrease in cervical cancer and increase in UNOS may be caused by the revision of the International Classification of Diseases (ICD). Therefore, the proportion of UNOS declines should be considered to examine changes in cervical cancer mortality. Furthermore, to obtain an accurate calculation for long-term analysis, Lloroa suggested that the proportion of UNOS and uterine cancers should combined to determine cervical cancer mortality rates [28].

However, this assumption and concept do not need to be considered in this study. Furthermore, the above conditions indicate that the study design included analysis of long-term trends and more than one version of ICD codes. In the studies conducted by Lloroa and Levi, the periods analyzed were 1955 to 1995 and 1960 to 1998, and they included 4 versions (ICD-7^th^ to 10^th^) and 5 versions (ICD-6^th^ to 10^th^) of ICD codes. To the best of our knowledge, the different versions of ICD codes were developed through diagnosis and treatment. Thus, we can infer that the old version may be the primary cause of the assumption. Based on this condition, we calculated the cervical cancer mortality trends for 1981 to 2010, but we only included two versions of ICD codes. However, the death registration system in Taiwan cited ICD-9^th^ for death certificates for 1981 to 2008, and ICD-10^th^ for 2008 to the present. Therefore, the effects of various versions of ICD codes are the lowest in this study.

The accuracy of cause-of-death coding in Taiwan appears to vary according to the type of disease[32]. Disagreements between the reviewer and the original coder included disagreements regarding the nomenclature, inappropriate judgments of cause relationships, and incorrect interpretation of the International Rule for Selecting the Underlying Cause of Death, and Modifying the Selected Underlying Cause of Death. The cause-of-death data used in this study was analyzed in 1994 and published in 2000. Therefore, the early cause-of-death data may influence the accuracy of the study results; this is the inevitable study limitation.

The quality indicators included the percentage of morphologically verified cases (MV%). The percentage of death-certificate-only cases (DCO%) between 1980 and 2009 show steady improvements in the quality of Taiwan Cancer Registry. The MV% increased from 82.4% between 1980 and 1984 to 89.05% between 2005 and 2009. The DCO% declined from 28.8% between 1985 and 1989 to 1.4% between 2005 and 2009.

## Conclusions

The results of this study support the effectiveness of the screening program through analysis of the results of the APC model and other standard statistic techniques. For both older and younger women in Taiwan, the screening program provided positive effects by reducing mortality rates; however, it also provided a delayed effect among elderly women. The reliability of supplementary measures following screening using Papanicolaou tests may be significant contributors. However, additional related studies with demographic analyses of the initial screening program implementation delay pattern are required for clarification in future research.

## Abbreviations

PT: Papanicolaou technique; HPV: Human papillomavirus; VIA: Visual inspection of the cervix with 5% acetic acid; APC: Age-period-cohort; ICD: International classification of diseases; AIC: Akaile information criterion; WHO: World health organization; ASMR: Age-standardized mortality rate; UNOS: Cancer of the uterus at an unspecified site.

## Competing interests

The authors declare that they have no competing interests related to this manuscript.

## Authors’ contributions

SSY participated in the design, data analysis, research implementation, results interpretation, and manuscript writing. LYP participated in the design, research implementation, and manuscript drafting and editing. JYH participated in the data analysis. DPJ and CCH contributed to the searching and management of the reference list. All authors have read and approved the final manuscript.

## Pre-publication history

The pre-publication history for this paper can be accessed here:

http://www.biomedcentral.com/1471-2458/13/13/prepub
